# 4D Bragg Edge Tomography of Directional Ice Templated Graphite Electrodes

**DOI:** 10.3390/jimaging6120136

**Published:** 2020-12-11

**Authors:** Ralf F. Ziesche, Anton S. Tremsin, Chun Huang, Chun Tan, Patrick S. Grant, Malte Storm, Dan J. L. Brett, Paul R. Shearing, Winfried Kockelmann

**Affiliations:** 1 Electrochemical Innovation Lab, Department of Chemical Engineering, University College London (UCL), London WC1E 7JE, UK; ralf.ziesche.16@ucl.ac.uk (R.F.Z.); c.tan@ucl.ac.uk (C.T.); d.brett@ucl.ac.uk (D.J.L.B.); p.shearing@ucl.ac.uk (P.R.S.); 2 The Faraday Institution, Quad One, Harwell Science and Innovation Campus, Didcot OX11 0RA, UK; patrick.grant@materials.ox.ac.uk; 3 Diamond Light Source Ltd., Harwell Science and Innovation Campus, Didcot OX11 0DE, UK; malte.storm@diamond.ac.uk; 4 Rutherford Appleton Laboratory, Science and Technology Facilities Council (STFC), ISIS Facility, Harwell OX11 0QX, UK; 5 Space Science Laboratory, University of California, Berkeley, CA 94720, USA; astr@berkeley.edu; 6 Department of Materials, University of Oxford, Oxford OX1 3PH, UK; ann.huang@kcl.ac.uk; 7 Department of Engineering, King’s College London, London WC2R 2LS, UK

**Keywords:** time-of-flight, energy-resolved imaging, Bragg edge imaging, neutron tomography, Li-ion battery, directional ice templated electrode

## Abstract

Bragg edge tomography was carried out on novel, ultra-thick, directional ice templated graphite electrodes for Li-ion battery cells to visualise the distribution of graphite and stable lithiation phases, namely LiC_12_ and LiC_6_. The four-dimensional Bragg edge, wavelength-resolved neutron tomography technique allowed the investigation of the crystallographic lithiation states and comparison with the electrode state of charge. The tomographic imaging technique provided insight into the crystallographic changes during de-/lithiation over the electrode thickness by mapping the attenuation curves and Bragg edge parameters with a spatial resolution of approximately 300 µm. This feasibility study was performed on the IMAT beamline at the ISIS pulsed neutron spallation source, UK, and was the first time the 4D Bragg edge tomography method was applied to Li-ion battery electrodes. The utility of the technique was further enhanced by correlation with corresponding X-ray tomography data obtained at the Diamond Light Source, UK.

## 1. Introduction

Bragg edge neutron imaging combines direct probing of a sample in real space with the collection of structural information by taking advantage of the coherent scattering cross-section of crystalline materials for slow neutrons [1,2,3]. The first spatially and energy resolved neutron transmission imaging experiments were undertaken around two decades ago to study the crystal structures of materials using a continuous reactor source [4,5] (with a good spatial but poor energy resolution) and a pulsed neutron spallation source [6,7,8] (with a poor spatial but good energy resolution), the latter being based on time-of-flight (ToF) methods. The experimental techniques converged with regard to spatial and energy resolutions with the introduction of double-crystal monochromators (DCM) and high spatially and temporally resolved pixel detectors at pulsed sources [9]. Bragg edge imaging at both continuous and pulsed neutron sources has been developed to provide an essential tool for materials scientists, for mapping structural variations such as strain, texture, phase and composition [10,11] and phase transformations [12] in, for example, engineering materials.

The overwhelming majority of Bragg edge studies collect two-dimensional (2D) data for one or a small number of sample orientations, where the acquisition time of one 3D energy resolved radiograph (2D spatial + energy) is typically of the order of minutes or hours, in order to scan the incident energy with a DCM or obtain meaningful count levels in hundreds or thousands of ToF channels. Long counting times for such Bragg edge radiographs often preclude the collection of tomography data, which is the case at the medium-flux neutron beamline IMAT at the pulsed spallation source ISIS, UK. The first 4D (i.e., energy and 3D spatially resolved) Bragg edge tomography study (BET) by Woracek et al. [13] used a DCM arrangement to study the phase transformation and distribution of austenite and martensite in TRIP steels. The first 4D BET experiments at a pulsed neutron source were demonstrated on a quenched steel cylinder by Watanabe et al. [14] and on a multi-material test sample by Carminati et al. [15]. They showed that in principle, 4D BET is a powerful tool to study crystallographic variations and transformations introduced by mechanical stress or chemical reactions.

Bragg edge imaging of the materials used in Li-ion batteries (LIB) was performed by Butler et al. [16] who studied the stepwise evolution of the LiC_6_ Bragg edge of graphite (used as the LIB anode material) during charging of a prismatic cell using a LiCoO_2_ (LCO)-based cathode. Later, Kino et al. [17,18] reported the use of Bragg edge imaging for studying crystallographic transitions in the cathode and anode of an 18650 LIB at different states of charge (SoC). Both studies captured the crystallographic phase transitions during dis-/charging of the cells by radiography, i.e., yielding path-integrated information through the Li-ion battery cell.

Here, we report 4D BET measurements on ultra-thick, electrochemically lithiated directional ice template (DIT) graphite LIB electrodes at various states of lithiation. The experiment was carried out on the IMAT beamline at the ISIS pulsed neutron source, UK. The aim of the work was to demonstrate that 4D BET was able to resolve different lithiation states inside the graphite-based electrodes locally at the voxel scale. The lithiation state of graphite is marked by both a change in the lattice d-spacings, mainly along the c-axis of the graphite structure, and by the wavelength-resolved reconstructed attenuation coefficient. Three phases can be detected: graphite, stable LiC_12_ and LiC_6_ phases. Electrodes made by the DIT technique provided thicknesses of more than one millimetre that were well-suited to Bragg edge analysis with a Microchannel Plate/Timepix detector [19], which has a pixel size of 55 µm. Complementary X-ray tomography was collected on the imaging branch line of the I13L beamline at the Diamond Light Source (DLS).

## 2. Experimental

### 2.1. Directional Ice Templated Electrodes

State-of-the-art LIB electrode thicknesses are tailored to between 20 and 100 µm, to balance volumetric energy density (thick electrodes) and power density (thin electrodes). With increasing electrode thickness, Li ion-deficient regions may develop inside the electrode. Further, at high power (several charge/discharge cycles per hour), the electrode/electrolyte regions that are furthest away from the macroscopic separator between anode and cathode develop a relatively low local Li ion concentration that results in capacity loss. To reduce this heterogeneity in local Li-ion concentration that develops at higher dis-/charge rates, thinner electrodes are used to shorten the Li-ion diffusion path but, unfortunately, this increases the volume fraction of inactive components in the LIB, such as current collectors and separators, and so reduces the overall cell volumetric capacity. The tendency to build up unhelpful, steep through-thickness gradients in Li ion concentration is also exacerbated by the highly tortuous pore network of the electrode.

As an alternative to thinner electrodes to preserve useful power performance, pore network engineering can be beneficial, especially by reducing the pore tortuosity in the critical through-thickness direction. Oriented, through-thickness pore channels can be provided by directional ice templating originally developed for ceramics [20] and then adapted for LIB electrodes [21]. The DIT technique provides highly elongated, channel-like pores in the electrode structure at the micro-scale and may also provide a relatively high electrode–electrolyte interface area. Together, these features promote improved Li-ion supply throughout the electrode thickness, even with an electrode thicknesses of over 1 mm, leading to very high areal and gravimetric capacity [22].

For the experiment, four 6 mm and one 12 mm diameter DIT graphite-based electrodes typical of a LIB were manufactured from a homogeneous aqueous suspension containing: active graphite particles (BFC-18, China), electrically conductive carbon black nano-particles and a sodium carboxymethyl cellulose (CMC) binder at a weight ratio of 25:1:1. This mixture was directionally and rapidly frozen in an in-house DIT apparatus using a Cu cold finger immersed on one end in liquid N_2_, at a cooling rate of approximately 0.1·K·s^−1^ measured by thermocouples at various locations on the apparatus. A more detailed description of the electrode making process is presented elsewhere [22] for a DIT LCO cathode for a LIB. Table 1 lists the dimensions of the manufactured electrodes.

The DIT electrodes were then transferred into an Ar-filled glovebox. The four 6 mm diameter electrodes were mounted in a Li-metal vs. graphite half-cell using 1/4″ diameter Swagelok-type PFA (perfluoralkoxy alkane) cells, according to [23]. The cells use two steel pins as positive and negative electrode current collectors, and between them, the half-cell comprised a Li-metal anode (MTI Corporation, Richmond, CA, USA), a polypropylene film separator (Celgard 2400, Celgard LLC, Charlotte, NC, USA) and the DIT graphite cathode, all immersed in a 1.0M LIPF_6_ in EC:EMC (50/50, v/v) electrolyte solution. The Li foil electrode and the separator were punched as 6 mm discs before assembling the cell inside the glove box. The cell was sealed by screwing 1/4″ PFA Swagelok (Swagelok Company, Solon, OH, USA) straight union connectors against the steel pins.

Outside the glovebox, the cells were connected to a Gamry Instruments (Warminster, PA, USA) 1000E potentiostat and discharged to 33%, 66%, 70% and 100% lithiation at a constant discharge current of 100 µA. Table 1 lists the electrode mass, theoretical electrode capacity [24], discharge capacity, estimated lithiation state and expected predominant phase for each electrode. To estimate the lithiation state, it was assumed that 90% of the electrode weight comprised active graphite participating in the lithiation reaction.

After the discharging process, the cells were disassembled in a glovebox to recover the lithiated electrodes. All electrodes showed a colour change due to the Li intercalation: from black in the pristine state, to gold for the fully lithiated LiC_6_ state [25]. To facilitate neutron transmission, any remaining electrolyte was removed as incoherent neutron scattering by H would be a source of noise, blurring the neutron images and hampering a quantitative analysis. The vacuum-dried 6 mm diameter electrodes were assembled into a single 1/4″ Swagelok cell, with 0.25 mm thick Cu discs between them to separate the electrodes and to prevent Li diffusion between them. Separately, an uncycled, pristine 12 mm diameter DIT graphite electrode was vacuum-dried to remove condensed water and assembled in a 1/2″ Swagelok cell. The use of the Swagelok cells throughout was to protect the electrodes from any moisture and spontaneous delithiation by reaction with air.

### 2.2. Bragg Edge Tomography

**Figure 1:**
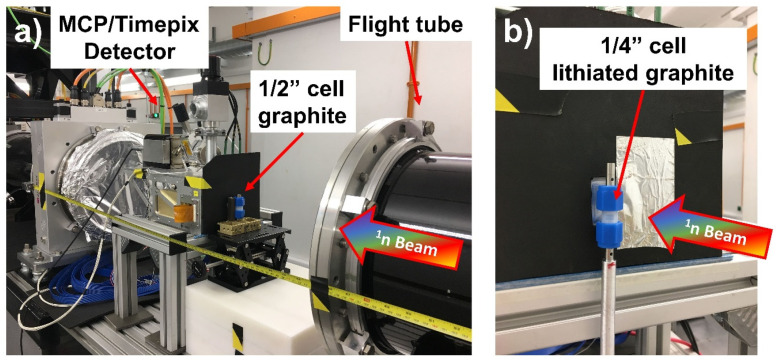
Experimental set-up at the “5 m position″ on IMAT. (**a**) MCP/Timepix detector installed 5.4 m downstream from the pinhole collimator, and a 1/2″ Swagelok cell in front of the detector; (**b**) 1/4″ Swagelok cell mounted on an Al rod on top of a rotation stage for tomography.

The neutron experiments were carried out at the IMAT neutron imaging and diffraction beamline on target station 2 (TS2) at the ISIS pulsed neutron source [26,27,28], which operates at 10 Hz. IMAT uses a liquid H_2_ moderator at 18 K to shift neutron energies to a cold spectral range. For BET, a new sample position was used, at approximately 51 m from the moderator and 5 m downstream of a neutron guide and “pinhole” collimator. At this position, a four times higher neutron flux is available compared with the regular position at 10 m from the pinhole, for the same pinhole size and a relaxed spatial resolution due to the smaller L/D ratio. For the utilised pinhole size of D = 80 mm and L/D = 62.5, the integral neutron flux was approximately 1 × 10^8^ n·cm^−2^·s^−1^. The sample was mounted on a rotation stage (Physik Instrumente, Karlsruhe, Germany) that was placed on a manual lifting table, just in front of a microchannel plate (MCP)/Timepix detector (Figure 1a). The MCP detector consisted of a stack of MCPs, with a neutron-sensitive MCP on the beam-facing side, in front of a 2 × 2 array of Timepix charge readout chips with 256 × 256 pixels each, and 55 µm pixels resulting in a field of view (FoV) of 28 × 28 mm^2^ [19,29]. In order to access the low-indexed Bragg edges of the different lithiated DIT graphite electrode LiC_x_ phases, the IMAT wavelength range was adjusted by using double-disk choppers [30] to provide neutron wavelengths between 2.5 and 8.3 Å corresponding to a ToF range of 32.5–122.5 ms. These settings enabled the observation of the first Bragg edges of pure graphite (002 at 6.72 Å), the stable LiC_12_ (002 at 7.02 Å) and the fully lithiated LiC_6_ phase (001 at 7.38 Å). The ToF range between two successive ISIS pulses (1/10Hz = 100 ms) was subdivided into four acquisition frames per neutron pulse; in other words, the MCP detector was read-out four times between pulses, to reduce deadtime effects due to event-overlaps [31]. This was important because of the higher than usual neutron dose on the “5 m position”. For each sample angle of a radiographic projection, a stack of 2685 ToF images was obtained corresponding to a narrow ToF bin of 20 µs and 40 µs below and above neutron wavelengths of 4.5 Å, respectively. The flight path (51.45 m) from the neutron source to the MCP detector was calibrated using data collected on a 10 mm ferritic Fe square-rod for which the positions of Bragg edges were known. The best spatial resolution given by the geometric blur was 20 mm/62.5 = 320 µm, for L/D = 62.5 and a sample-sensor distance of 20 mm.

A Bragg edge radiography stack was collected for reference from the graphite-only DIT (electrode-0), as shown in Table 1. The 1/2″ Swagelok cell was positioned close to the MCP detector for an exposure time of 1.5 h. For the combined set of four lithiated electrodes in the 1/4″ cell, one complete BET was collected from the cell mounted on top of an Al rod that was connected to the PI rotation stage and positioned close to the MCP detector (Figure 1b). The size of the neutron beam was set using beam slits to fully illuminate the sensitive 28 mm× 28 mm area of the MCP. Neutrons passed through the PFA casing of the Swagelok cell without significant attenuation. The sample cell was positioned in front of the left lower Timepix chip of the MCP, thus preventing sample images from crossing the neutron-insensitive gaps between the four Timepix chips for all sample rotations. The golden ratio (GR) scanning strategy [32,33,34] was used, which allows flexible termination of a tomographic scan. The tomography, scanned over an angular range of 180° with an exposure time of 1.5 h per projection, was stopped at 50 projections, yielding an effective pixel size of 150 µm and a spatial resolution of 300 µm for the 6 mm diameter electrodes. The angular step size between projections was up to 100° with dead-times of up to 15 s for the rotation stage movement, i.e., insignificant compared to the exposure time. For collection of a 1.5 h open-beam radiography stack, the sample was removed from the stage. 

The image stacks were “overlap-corrected″ as described by Tremsin et al. [31] and time-binned for 5 consecutive images of each image stack that resulted in 537 ToF bins per projection. Binning was required due to the low neutron count statistics in a space-time pixel, especially for the longer wavelength region between 6 and 8 Å, and to better match the intrinsic instrument wavelength resolution of ∆λ/λ of 0.4% at the longer wavelengths [35]. The resulting ToF increments after binning were 102.4 and 204.8 µs (0.00787 and 0.01575 Å) below and above 4.5 Å. Radiographs/projections were filtered, to remove outliers and dead pixels, and normalised using an open beam region (away from the sample) to correct for a slightly decreasing detector efficiency during the tomography scan. Finally, the sample image stacks were flat-field-corrected with respect to the open beam image stack. For the tomographic reconstruction of the 1/4″ Swagelok cell, the projections were cropped to the region of interest that included the sample.

As a first step, all time bins of a stack were combined to produce “white beam projections″ with high signal to noise ratio (SNR) and high contrast, to find the centre of rotation for the energy-resolved BET dataset. Then, 3D tomographs were reconstructed for each energy bin using the filtered-back-projection (FBP) algorithm of the ASTRA toolbox reconstruction library in Python [36,37]. The corresponding 537 tomographs, constituting a 4D reconstruction set, contained in each voxel the wavelength-dependent attenuation coefficients of each material component/structure in terms of Bragg edges.

### 2.3. X-ray Tomography

To analyse the structural development and porosity of the DIT graphite electrodes, X-ray micro-computed tomography (CT) was carried out on the Diamond Manchester Imaging branchline (I13-2) at the Diamond Light Source (DLS, UK) [38]. Due to the weak absorption contrast between the active graphite material and the carbon black binder, the electrodes were placed further away from the scintillator screen of the camera system to enhance the phase contrast caused by the refraction of the X-ray beam at the surfaces and interfaces of particles.

Each cell was mounted separately on the I13-2 rotation stage system consisting of a HUBER 1002 Goniometer Head (HUBER Diffraktionstechnik, Rimsting, Germany) on top of two perpendicularly mounted Newport MFA-PPD (Newport Corp., USA) linear stages on an Aerotech ABRT-260 (Aerotech Inc., Pittsburg, PA, USA) rotation stage. The two linear stages helped to align the sample with the rotation axis in the centre of the field of view of the camera system, incorporating objective lenses with different magnifications. For both tomographies (the 1/2″ and 1/4″ cells), a 500 µm thick CdWO_4_ scintillator foil on the entrance of a 2× objective lens (Olympus PlanApoN 2x), mounted ahead of a 2× tube lens providing 4× total magnification, was selected. The camera was protected from the direct X-ray beam by utilising a mirror at 45° that reflected visible light to the CMOS camera chip placed 90° to the incident beam. The usable FoV was 4.2 × 3.5 mm with an effective pixel size of 1.625 µm. For the imaging process, a pink beam X-ray energy spectrum in the range 20–25 keV was used, with graphite and Al filters. Due to the large diameter of the 12 mm DIT electrode, tomographs from only a selected internal volume of the electrode were collected. The 1/2″ Swagelok cell was aligned with the cell centre in the centre of the rotation axis of the sample stage and a sample to scintillator distance of 215 mm to enhance phase contrast. One tomogram was collected over an angular range of 180° with 1800 steps and an exposure time of 20 ms per projection in flight scan mode, where projections were continuously recorded during sample rotation. The four smaller lithiated electrodes were scanned in “double FoV” mode with the rotation axis close to the left side of the detector edge that extended the FoV to approximately twice the size in the horizontal direction, thus allowing a scan of the whole electrode. Accordingly, the angular scanning range was extended to 360° with 3600 projections. The projections were corrected using open beam images and the dark current of the camera. For tomographic reconstruction, the ASTRA toolbox [36,37] and Tomopy [39] reconstruction libraries in Python were used. In the case of the “double FoV” measurements, projections in the range 0° to 180° were overlapped with the projections from 180° to 360° to generate a dataset from 0° to 180° with double projection width.

## 3. Results and Discussion

### 3.1. Coral-Like DIT Electrode Structure

**Figure 2:**
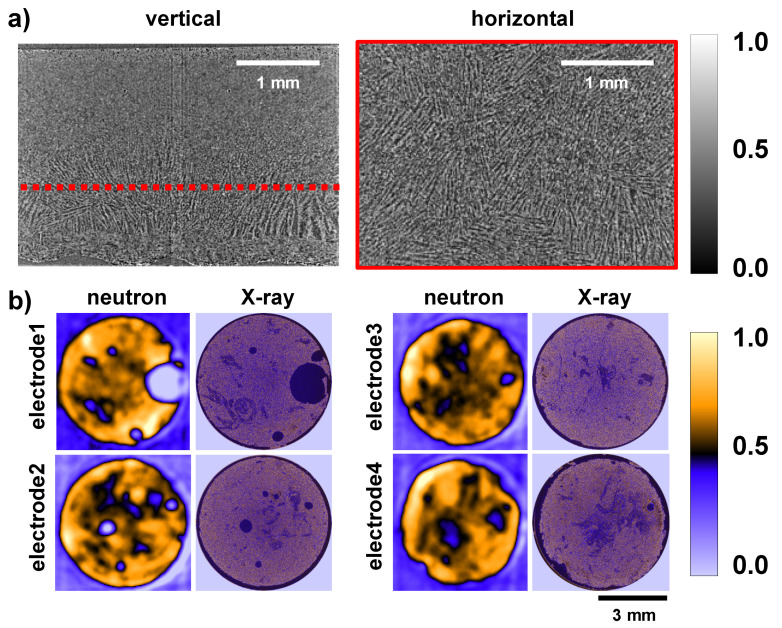
Orthogonal slices of the reconstructed X-ray tomogram of the 12 mm diameter DIT graphite electrode and comparison with the polychromatic neutron and X-ray attenuation of the lithiated DIT graphite electrodes. (**a**) Vertical and horizontal slices of the 12 mm graphite electrode showing the coral-like pore structure with larger radii and longer branches in the lower electrode section in the vertical slice. (**b**) Horizontal slices of the four 6 mm diameter electrodes, indicating (for the neutron data) the attenuation-based distribution of the graphite, LiC_12_ and LiC_6_ phases. Low attenuating regions are bright blue and blue; higher attenuating regions are yellow. For comparison, the X-ray images show the graphite density distribution at the same electrode height.

Figure 2a shows vertical and horizontal orthogonal slices of the reconstructed X-ray tomogram of the ultra-thick 12 mm diameter DIT graphite electrode. The tomography displays the whole electrode height of ca. 2.5 and 3.95 mm wide section from a central region of the electrode. Clearly visible is the aligned, coral-like electrode structure induced by the directional freezing. There were wider and longer bacillar features in the lower electrode region and a decreasing size of features in the upwards direction. The slices show the active graphite particles and carbon binder, and the air-filled channels as white and black features, respectively. In a cell assembly, these pore channels are filled by the electrolyte. The horizontal slice shows a slightly magnified electrode section to illustrate the aligned channel features formed by the directional freezing that pushed the solid components (graphite and carbon) of the suspension into the shrinking regions between the growing ice crystals. The growth of the ice started in the lower image section, visible in the vertical slice, and progressed towards the upper surface.

Similar coral-like structures are visible in the thinner, 6 mm diameter lithiated DIT graphite electrodes. In contrast to the larger cell, they displayed somewhat more inhomogeneous structures with a few relatively large air bubbles, particularly in electrode-1 and electrode-2, with bubble diameters up to 1.5 mm (Figure 2b). The remaining two electrodes (electrode-3 and electrode-4) had a more homogeneous structure, with only a slight variation in the spatial dispersion of the active graphite particles and the binder. It should be noted that these electrodes represent the first proof of concept DIT electrodes containing graphite as the active electrode material, and it is anticipated that improved reproducibility and microstructural control could be achieved through ongoing refinement of the experimental method.

### 3.2. Bragg Edge Transmission Imaging

**Figure 3:**
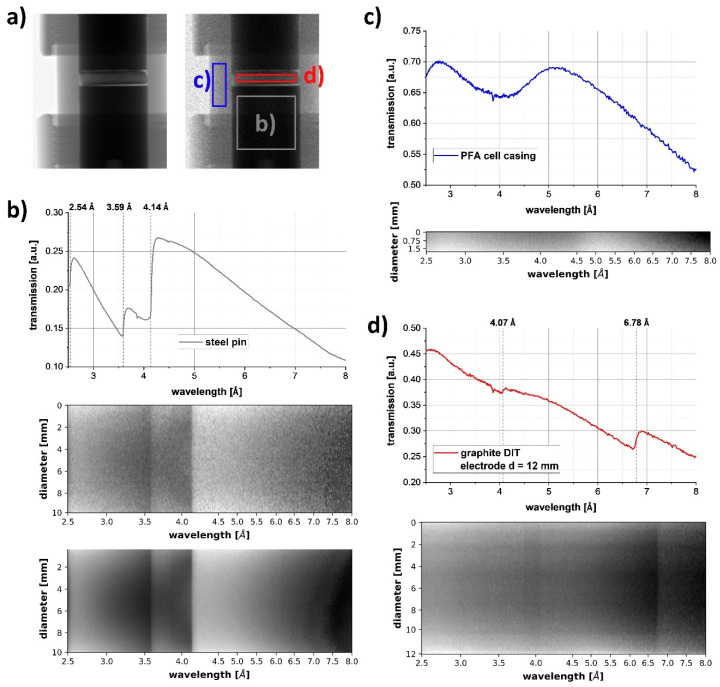
Bragg edge transmission radiography of the 12 mm diameter DIT graphite electrode inside a 1/2″ Swagelok cell. (**a**) Polychromatic neutron radiograph (left) and a monochromatic radiograph at 2.6 Å (right). Coloured boxes mark regions of interest for the transmission curves for the steel pin (**b**), the PFA region (**c**), and the electrode (+ PFA housing) (**d**). Corresponding wavelength-resolved transmissions for a single pixel row and for summed-up transmissions over multiple rows are displayed for the same regions of interest.

Figure 3a shows cropped radiographs from the larger 1/2″ Swagelok cell containing the larger 12 mm diameter and 2.5 mm thick DIT graphite electrode. The left image shows the transmission contrast of the polychromatic neutrons with all principal cell components visible, including the steel pins/current collectors (dark) above and below the electrode, and the PFA union connector around the electrode and pins with fittings screwed at the union endings on both sides for sealing. The radiograph on the right shows the cell imaged with a narrow, almost monochromatic wavelength band at approximately 2.6 Å. Due to the lower number of neutrons in the narrow wavelength bin, the radiograph is noisier than the white-beam radiograph.

The Bragg edge spectra for three regions of interest are displayed in Figure 3b–d. The steel pin regions have Bragg edges at 2.54, 3.59 and 4.14 Å (Figure 3b) corresponding to the (022), (002) and (111) planes of the face-centred cubic (fcc) lattice. Figure 3b shows the wavelength-resolved transmission intensity for a single 55 μm pixel row and the transmission intensity averaged over a large number of pixel rows, respectively, illustrating the Bragg edge contrast as a function of wavelength for the pin. The PFA transmission intensity plot in Figure 3c exhibited a minimum of intensity at ~4 Å due to short-range-order. Figure 3d displays the transmission intensity through the DIT graphite electrode wavelength-resolved for the region marked by the red box in (a). In the transmission spectrum, the graphite signal and PFA spectrum are superposed. Two graphite Bragg edges, (01-1) at 4.07 Å and (002) at 6.78 Å, are visible. This indicated that Bragg edge imaging can be applied to resolve the crystal structure of graphite within the DIT electrode.

The neutron-path-averaged attenuation coefficients in a radiograph can impede quantitative analysis if the material compositions, sizes and density of components are uncertain, even though the Bragg edge information helps to deconvolute some of this complexity. A wavelength-resolved 4D BET reconstruction was the next step to further separate structure and density-specific attenuation coefficients, locally resolved in single voxels.

**Figure 4:**
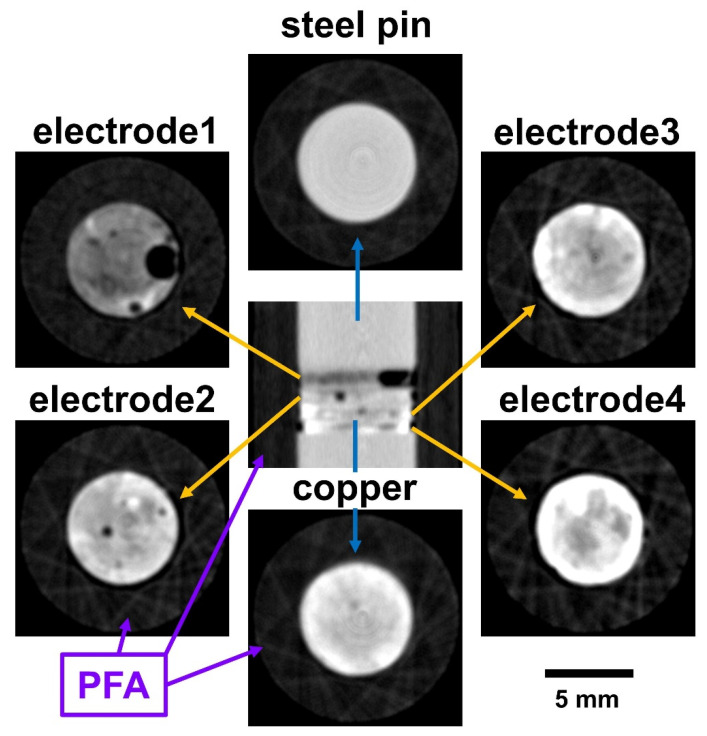
Polychromatic neutron tomographs of the 1/4″ Swagelok cell: the central image shows a vertical orthogonal slice through the cell. Horizontal slices of the different cell components are shown including the steel pins, the Cu spacers between the four different lithiated 6 mm diameter DIT graphite electrodes and the surrounding PFA casing. Due to the modest spatial resolution and the small number of projections, artificial contrast variations and other artefacts may arise.

Figure 4 shows reconstructed slices of the cropped white-beam neutron tomography dataset from the 1/4″ Swagelok cell, which contained the four lithiated 6 mm diameter DIT graphite electrodes sandwiched between the two steel pins above and below, and with the four electrodes separated by 0.25 mm thick Cu spacers. The slice of the steel pin displayed a relatively homogeneous intensity whereas the Cu spacer had some structured contrast, probably due to texture variations and/or due to the moderate spatial resolution in the range of the thickness of a spacer. Further, due to the relatively small number of projections, the reconstruction of the surrounding PFA housing exhibited some star-like artefacts. Larger structural features of the electrodes, such as the air bubbles in electrode-1 and electrode-2, were clearly visible. Moreover, significant contrast differences between the individual electrodes were detectable at 33% lithiation and increasing grey values (i.e., attenuation) for electrode-2 and electrode-3 at 66% and 100% lithiation, respectively. Electrode-4 was 70% lithiated but showed a higher attenuation than the 100% lithiated electrode-3. This may be due to density differences such as differences in the overall porosity fraction of electrodes, as well as possible errors in ensuring the lithiation state. For instance, the Li density appeared higher at the electrode edges than in the middle. Higher local Li concentrations should produce higher attenuation, which was not the case. This aspect indicates that a quantitative determination of the Li concentration or SoC only by the grey level change was not possible. The uncertainty is further emphasised in Figure 2b, which compares white-beam neutron slices with corresponding X-ray slices. The image colours display a low attenuation as bright blue (representing air) or dark blue/black, whereas yellow represents higher attenuation, e.g., LiC_12_ and/or LiC_6_. For the neutron images, the contrast was optimised for each electrode to allow a qualitative analysis of regions where there was a relatively high graphite-phase density and/or a higher level of lithiation. Each electrode showed strong fluctuations of attenuation across regions of interest, with a low attenuation indicating air bubbles, especially in electrode-1 and electrode-2, and notably in the middle regions of the electrodes. The graphite particle density was greater at the electrode edges.

The X-ray images in the right columns of Figure 2b provide an insight into the local graphite (or pore) fraction because the Li concentration did not affect the X-ray attenuation. The local graphite density distributions from X-ray CT were consistent with the low attenuation regions from the neutron images, which leads to the conclusion that the principal attenuation mechanism was local density variation due to changes in the local pore fraction and was not due to variations in the lithiation state.

**Figure 5:**
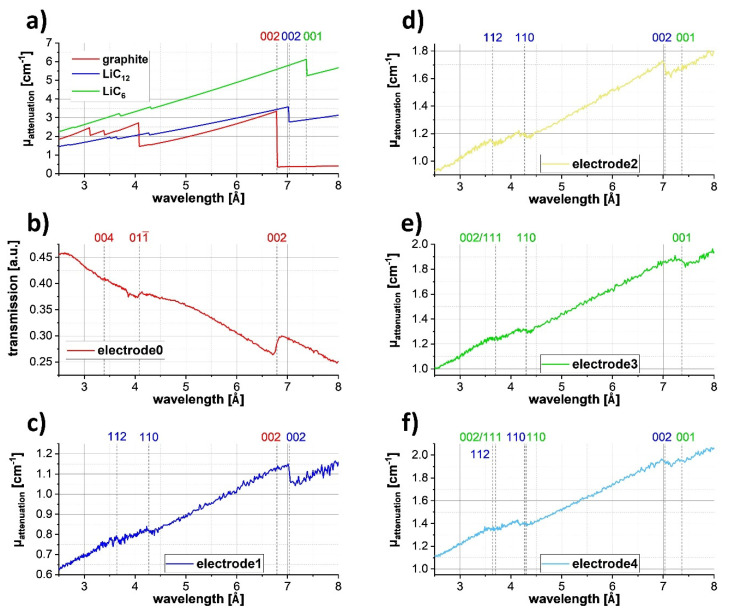
Identification of crystallographic lithiation phases using Bragg edges. (**a**) Calculated attenuation spectra for graphite (red), LiC_12_ (blue) and LiC_6_ phases (green). The dashed vertical lines indicate Bragg edge positions. (**b**) Transmission spectrum from the radiograph of the 12 mm diameter pristine DIT graphite electrode, identifying the graphite phase with dominant (002), (01-1) and (004) Bragg edges (mixed with the PFA signal). (**c**) Reconstructed wavelength-resolved attenuation coefficients of mixed graphite and LiC_12_ phases in the 33% lithiated electrode-1; (**d**) LiC_12_ for electrode-2 at 66 % lithiation; (**e**) LiC_6_ for electrode-3 at 100% lithiation; (**f**) mixed LiC_12_ and LiC_6_ at 70% lithiation in electrode-4. For each 6 mm diameter electrode, the attenuation curve was extracted for a representative horizontal slice and averaged from a region of interest within the slice.

Diffraction-based imaging techniques such as BET overcome the local density problem as it provides extra information by resolving the specific lithiation phases and structures. To determine the lithiation state of each electrode, the wavelength-resolved neutron attenuation coefficients were plotted in Figure 5 for a large region of interest, comprising slices taken from the middle section of the electrodes. Figure 5a shows the calculated attenuation coefficients from nxsPlotter [40] using CIF-files for graphite [41], LiC_12_ and LiC_6_ [42] phases. There was an increasing attenuation coefficient with increasing Li content in the graphite (red) to LiC_12_ (blue) and LiC_6_ (green). Marked changes in the attenuation curves were at approximately 7 Å, where the coherent scattering contribution of the graphite, LiC_12_ (002) and LiC_6_ (001) planes disappeared, i.e., at 6.79, 7.03 and 7.37 Å for graphite, LiC_12_ and LiC_6_, respectively. The Bragg edges shifted towards longer wavelengths because of increased interatomic d-spacings along the c-axis of the graphite during Li intercalation. The transmission curve of the pristine DIT graphite electrode-0 (Figure 5b mixed with the PFA signal) had a relatively intense (002) graphite Bragg edge at 6.79 Å in accordance with the calculated data using nxsPlotter. Additionally, there were two smaller edges at 4.08 and 3.39 Å ascribed to (01-1) and (004) lattice planes, respectively. The observed Bragg edge height ratios for electrode-0 were found similar to the expected ratios for randomised grain orientations, thus indicating absence of texture or weak texture. Note that the slope and Bragg edge features in the transmission curves are inverted when compared with the attenuation coefficients due to Beer–Lambert’s law. During lithiation of graphite, metastable phases are formed, such as LiC_24_ and LiC_18_, which disappear after a relaxation time and are replaced by the more stable and energetically favourable graphite and LiC_12_ phases [43]. Due to the time elapsed between electrode production and the neutron experiment, the electrodes showed only one or a mixture of the three stable phases: graphite, LiC_12_ and LiC_6_.

The reconstructed attenuation coefficient for the 33% lithiated electrode-1 exhibited LiC_12_ Bragg edges in the relaxed state, indicated by the strong (002) Bragg edge at 7.03 Å (Figure 5c). Two further edges were detected: (110) at 4.27 Å and (112) at 3.64 Å. Although not easy to identify by eye, there was a slight change in the slope between 6.7 and 7.0 Å due to the presence of a (002) graphite Bragg edge, which agreed with the expected mix of graphite and LiC_12_ before 50% lithiation is reached. At ~66% lithiation, only the (002) Bragg edge of LiC_12_ was resolved for electrode-2, as shown in Figure 5d. The expected mix of the LiC_12_ and LiC_6_ phases was not readily resolved because of the high noise levels in the data. Nonetheless, at 100% lithiation, a single LiC_6_ phase was resolved in Figure 5e for electrode-3, indicated by the (001) Bragg edge at 7.37 Å. There were also smaller (110) and overlapping (002)/(111) Bragg edges at 4.30 and 3.70 Å, respectively. There were mixed phases in electrode-4 ~70% lithiated, with Bragg edges of LiC_12_ and LiC_6_ shown in Figure 5f.

The attenuation curves for the DIT electrodes and for the lithiation phases in Figure 5 were approximately two to three times lower than the calculated attenuation coefficients. This discrepancy could arise due to sometimes high and inconstant porosity, as previously noted, while recognising 20–40 vol% porosity in the electrodes is a required feature of a useful LIB. Further, the similarity of the attenuation coefficients for different lithiation states (e.g., for electrode-3 and electrode-4 in Figure 5e,f) may relate to local variations in the graphite and LiC_x_ fractions, despite averaging.

**Figure 6:**
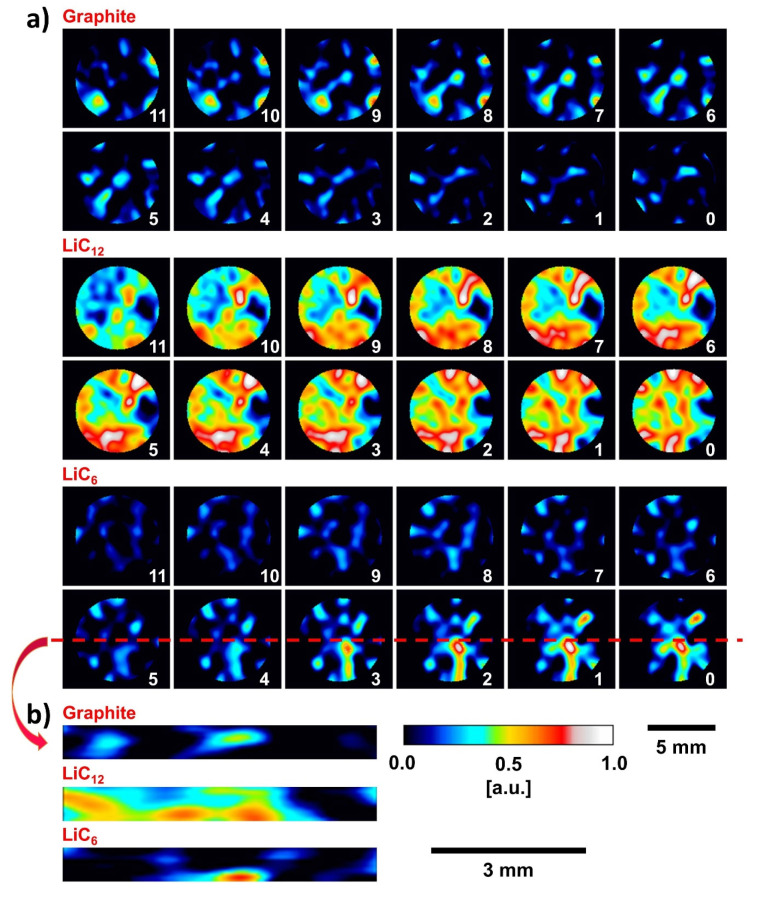
Maps of Bragg edge heights of graphite, LiC_12_ and LiC_6_ phases for (**a**) horizontal and (**b**) vertical slices for electrode-1 at ca. 33% SoC. The plots represent a 660 µm height region in the middle section of the electrode with a voxel size of 55 µm. The slices show an inhomogeneous distribution of the phases where black/blue represent the absence or a low proportion of the lithiation phase and red/white a high proportion. The horizontal slice number 0 was closest to the lithium-metal counter-electrode, i.e., with increasing slice number, the distance from the Li-metal electrode increased. The vertical slices show the phase distribution as an orthogonal slice through the electrode centre marked in (**a**). A higher lithiation state (LiC_6_) was detected close to the Li-metal electrode.

Quantitative data analysis can be realised by fitting the Bragg edges to an analytical function, as described by Tremsin et al. [44] for radiographic measurements. The approach involves analysing the transmission spectra calculated from reconstructed attenuation coefficients; see Figure 5b-f for comparison. The position and width of the edges were determined prior to the mapping process using a large region of interest. The (002)/(001) Bragg edges of the pixels of the reconstructed slices were fitted sequentially, whereby the low SNR issue was addressed by using a running average of macro-pixels of 20 × 20 pixels and a step size of 55 μm. Four parameters were fitted for each Bragg edge and each macro-pixel: baseline and slope before the Bragg edge; Bragg edge height; and slope beyond the Bragg edge (at larger wavelengths). The fitted Bragg edge heights indicated the phase fractions whereby the slopes beyond the (002) and (001) Bragg edges, respectively, were affected by Li absorption. Figure 6 shows maps of the best-fit Bragg edge heights for electrode-1 for 12 inner slices representing a 660 µm thick region of the electrode. The three maps in Figure 6a show the local distribution and relative density of the three lithiation states for graphite (002), LiC_12_ (002) and LiC_6_ (001). Black/blue represents the absence or a low proportion of the phase, and red/white indicates a high proportion, respectively. The maps indicate inhomogeneous distributions of the phases across the electrode. This heterogeneity may again be due to the inhomogeneous electrode porosity and/or the variation/dispersion in the graphite particle diameters. The horizontal slices in Figure 6a marked “0″ were closest to the Li-metal electrode, with increasing distance for the following slices, respectively. There were different proportions of graphite and LiC_6_ phases over the electrode height: in general, there was a higher lithiation state near to the Li source, and with unlithiated graphite on the opposite side, furthest away. Figure 6b shows vertical orthogonal slices of the Bragg edge height maps visualising the spatial distribution of the lithiation phases across the electrode height. A further analysis of the Bragg edge heights will allow quantification of the lithiation phases. The use of other spectral regions, particularly past the first Bragg edge, will also be investigated for the quantification of Li concentration and porosity.

## 4. Conclusions

Energy-resolved BET is a useful tool to investigate the crystallographic lithiation states in relatively thick graphite electrodes and also allows the local SoC to be estimated. These wavelength-resolved tomographic reconstructions provide information such as structure and density-specific attenuation coefficients locally resolved on a voxel level, which is difficult to achieve by other techniques. The proof of concept study presented here also suggests that the major phase transitions can be tracked: from graphite to mixed graphite/LiC_12_ and LiC_12_/LiC_6_ phases, and then to the single LiC_12_ and LiC_6_ phases as the extent of lithiation increases. The mapping of the phases not only reveals the local crystallographic lithiation state but suggests that quantitative determination of the local Li concentration and the local electrode porosity may be possible. Nonetheless, the collected neutron datasets typically suffered from relatively poor spatial resolution of ~300 µm, rather noisy data and long exposure times for a single BET, which makes the technique less favourable than some methods that yield similar information such as neutron diffraction. However, in comparison with neutron diffraction, BET surveys a larger part of the device in 3D whilst having a smaller gauge volume (i.e., higher spatial resolution), which is important for in situ analyses. Compared with the more usual application of Bragg edge imaging to study engineering metals and alloys, BET investigations of lithiated graphite electrodes are challenged by the unfavourably strong neutron absorption of Li and because the distinctive Bragg edges of lithiated phases are observed at relatively long wavelengths, where the IMAT neutron flux is reduced considerably.

The new sample position used at IMAT is not yet optimized and further improvements in efficiency might be achieved, which may reduce exposure times and increase the signal to noise ratio. The next generation of MCP detectors based on Timepix-3 readout [45] will improve the duty cycle of the detector by avoiding losses due to the overlap effect, with additional gains due to a larger field of view and more efficient neutron sensitive MCPs. Moreover, upcoming installations of neutron imaging beamlines at high-brilliance spallation sources such as the European Spallation Source in Lund (ESS, Sweden) [46] promise an improvement in terms of spatial and temporal resolution down to 110 µm. Moreover, high flux neutron reactor sources such as HFR at the Institut Laue-Langevin (ILL, France) or the FRM-II (Heinz Maier-Leibnitz Zentrum, Garching, Germany) are able to produce fast wavelength scans using DCMs and will not so much be limited by the neutron flux and the pixel sizes of the cameras.

As a next step, more homogeneously structured DIT or other graphite electrodes will be analysed at different SoCs and throughout a charge/discharge cycle. The analysis tools will also be further developed to determine inter alia quantitative information of the crystallographic/lithiation phases, the Li concentration and porosity information at a higher spatial resolution. The quantitative distribution of Li with 3D resolution is, in principle, also possible from the same dataset.

The BET technique offers an analysis option that fits into a gap in spatial resolution between neutron diffraction (>500 µm) [3] and 3D XRD (<5 µm) [47,48], but with the high penetration of neutrons for sample dimensions far beyond the reach of XRD. With the projected improvements to BET capabilities described above, we anticipate that BET may provide an important new tool to resolve microstructural and crystallographic changes in operating batteries on a voxel level and be able to determine local lithiation states. Such information will be useful, for example, to help unravel the complex degradation mechanisms of Li-ion battery electrodes and could find wider applicability to other electrode materials, such as newer Ni-rich NMC (LiNi*_x_*Mn*_y_*Co_1−*x*−*y*_O_2_, *x* ≥ 0.5) cathode materials [49].

## Figures and Tables

**Table 1:** Dimensions, mass, theoretical capacity, discharge capacity, lithiation state and expected majority crystallographic phase of the DIT graphite electrodes.

Electrode N	Thickness (mm)	Diameter (mm)	Mass (mg)	Theoretical Capacity (mAh)	Discharge Capacity (mAh)	Lithiation State *)	Dominant Phase
Electrode-0	2.57	ca. 12	159.8	59.13	0.00	0%	Graphite
Electrode-1	0.81	6.1	12.33	4.56	1.52	33%	LiC_12_
Electrode-2	0.48	6.0	8.61	3.19	1.90	66%	LiC_12_
Electrode-3	0.53	6.1	8.36	3.09	2.81	100%	LiC_6_
Electrode-4	0.49	5.9	8.20	3.03	2.136	70%	LiC_12_, LiC_6_

*) at 90% of theoretical capacity.
